# Novice Chinese as a foreign language teachers’ identity construction in primary schools in New Zealand from positioning and affordance perspectives

**DOI:** 10.3389/fpsyg.2022.979803

**Published:** 2022-11-10

**Authors:** Peijian Paul Sun, Yawen Wang, Yanxing Lv, Zhishan Li

**Affiliations:** ^1^School of Foreign Languages, Xiamen Institute of Technology, Xiamen, China; ^2^Department of Linguistics, Zhejiang University, Hangzhou, China; ^3^School of International Education, Zhejiang University, Hangzhou, China; ^4^Graduate School, The Tianjin Juilliard School, Tianjin, China

**Keywords:** being positioned, self-positioning, affordance, novice teacher identity construction, Chinese as a foreign language (CFL)

## Abstract

While there has been an increase in research on Chinese as a foreign language (CFL) teachers’ identity worldwide, limited attention has been drawn to CFL teachers’ positioning and affordances to interpret their identity construction in an overseas context. To fill the gap, this study investigated seven novice CFL teachers’ identity construction as Chinese language teachers in primary schools in New Zealand from positioning and affordance perspectives. Retrospective semi-structured interviews were adopted to understand how the novice CFL teachers were positioned, how they positioned themselves, and what affordances they perceived to be influential to their Chinese language teacher identity construction. The findings showed that the novice CFL teachers’ identity construction was subject to the social, institutional, and individual levels of being positioned, self-positioning, and affordances. Specifically, (1) consistent self-positioning at the social, institutional, and individual levels could largely determine the novice CFL teachers’ identity construction; (2) inconsistency of identities between being positioned and self-positioning at the social, institutional, and individual levels might weaken the novice CFL teachers’ identity construction; (3) affordances as opportunities at the social, institutional, and individual levels could strengthen the novice CFL teachers’ identity construction, whereas affordances as challenges could not. The study concluded with implications and limitations to inform future research.

## Introduction

In an era characterized by internationalization and multilingualism, and in a global economy in which China has the second largest share of the world GDP, Chinese as a foreign language (CFL) learning has gained significant popularity around the world. Taking New Zealand as an example, Chinese has become the fastest-growing foreign language (L2) taught in both schools and universities (Education Counts, 2021). To support CFL teaching worldwide, the center for language education and cooperation of China (originally known as the Office of Chinese Language Council International or Hanban) recruits thousands of pre-service CFL teachers as volunteers each year to teach Mandarin in primary and secondary schools and institutions of higher education overseas (Sun, 2021).

Among the recruited volunteer CFL teachers, most of them are novice in overseas Chinese language teaching. In other words, they may have little experience in terms of how to fit themselves in a different educational system, how to handle relationships with students, teachers, and school leaders in a cross-cultural context, and how to exploit resources and affordances to adapt their teaching to the local students. All the above may influence novice CFL teachers’ identity construction as Chinese language teachers and thus their future commitment to this profession. Since teachers’ identity construction and formation is a continuous process of interpretation and reinterpretation of their personal and professional experiences entrenched in complex social contexts, it is necessary to take teachers’ living and teaching experiences in their situated contexts into account to examine their teacher identity development.

Teacher identity has emerged as a critical construct for examining teachers’ professional lives, teaching quality, teaching motivation, and career decision-making (Richardson and Watt, 2018). A significant number of studies have examined teacher identity from linguistic, sociocultural, and poststructuralist (or integrated) perspectives, with their participants mainly focused on M.A. TESOL students in the US and English as a foreign language (EFL) teachers in Japan (see Kayı-Aydar, 2019 for details). However, limited attention has been drawn to CFL teachers’ identity construction and development in Western educational contexts (Wang and Du, 2014). Specifically, there is a lack of understanding of how novice CFL teachers position themselves in a different sociocultural context, how these teachers are positioned in the context, and what affordances they perceive to be beneficial to their overseas Chinese language teaching. In other words, whether positioning and affordances contribute to novice CFL teachers’ identity construction as Chinese language teachers during teaching abroad is a question worth investigation.

The present study, therefore, examined novice CFL teachers’ identity construction as Chinese language teachers in primary schools in New Zealand from positioning and affordance perspectives. It was hoped that implications and insights could be drawn to inform schools and teacher training programs to better support novice CFL teachers’ teacher identity construction in an overseas context. The present study was guided by the following overarching research question (RQ).

RQ: How do novice CFL teachers’ lived and teaching experiences as mandarin language assistants contribute to their Chinese language teacher identity construction in primary schools in New Zealand?

## Literature review

### Teacher identity and theoretical lenses

Teacher identity refers to teachers’ understanding of themselves and their relationships with others in their situated communities. However, “teacher identity is not something that is fixed nor is it imposed; rather it is negotiated through experience and the sense that is made of that experience” (Sachs, 2005, p. 15). In effect, teacher identity is dynamic and multifaceted and can be subject to various factors such as context, experience, beliefs, and personal attributes (Richardson and Watt, 2018; Richards, 2021). Therefore, a more comprehensive interpterion of teacher identity can be defined as “teachers’ dynamic self-conception and imagination of themselves as teachers, which shifts as they participate in varying communities, interact with other individuals, and position themselves (and are positioned by others) in social contexts” (Yazan, 2018, p. 21).

To examine the complex and multifaceted nature of teacher identity, different theoretical lenses have been adopted over the decades, including the social identity theory (Tajfel, 1978), the theory of situated learning (Lave and Wenger, 1991), the concept of the image-text (Simon, 1995), the communities of practice framework (Wenger, 1998), positioning (Davies and Harré, 1990), and affordance (Gibson, 1979, 2014). Given that each theory in isolation has its own limitations, recent research calls for a multiple theoretical approach to gaining a richer understanding of teachers’ identity (Varghese et al., 2005; Beauchamp and Thomas, 2009). Among the above theoretical lenses, positioning and affordance have rarely been adopted as analytical perspectives to examine novice CFL teachers’ identity construction as Chinse language teachers in an overseas context.

This study, therefore, employed positioning and affordance as an integrated analytical lens to understand novice CFL teachers’ identity construction as Chinese language teachers in primary schools in New Zealand. Specifically, interactive positioning (i.e., assigning positions to others or being positioned) and reflexive positioning (i.e., assigning positions to oneself or self-positioning), along with affordances (i.e., opportunities and challenges) that accompanied by positions, were collected for the examination.

### Teacher identity and positioning

Recent research in the field of language teacher education has witnessed a growing interest in the construction and formation of language teacher identity (Fairley, 2020; Goktepe and Kunt, 2021). For example, three major themes have been identified in a critical review of EFL teachers’ identity, including “identity development through social engagement,” identity struggle and conflicts under contextual constraints,” and “identity crisis with teacher attrition” (Yuan, 2019, p. 521). Apart from research on EFL teacher identity, recent attention has been drawn to CFL teachers’ identity.

In line with the EFL teacher identity literature, research on CFL teacher identity suggests that these teachers’ identity construction derives from their social engagements and linguistic practices in complex activity systems (Cross and Gearon, 2007; Lee, 2013; Gong et al., 2022). For example, Wang and Du (2014) studied immigrant Chinese teachers’ professional identity transformation in an intercultural context (i.e., Danish) and found that the professional identity of Chinese teachers was shaped both by their previous experiences of teacher-student relationship in the Chinese culture and their new experience as teachers in the Danish culture. Additionally, Li (2016) conducted a life-history narrative study of three CFL teachers who had over 20 years teaching experience. The study revealed that successful teacher identity construction in Western-based school contexts required “an effective blend of Eastern and Western cultural values and pedagogical practices” (p. 177).

Although previous research has evidenced the important role of social engagements and linguistic practices in shaping language teacher identity, findings of CFL teachers’ identity in cross-cultural contexts have been revealed to be divergent. On the one hand, some studies found that CFL teachers’ identity tended to be authoritarian and teacher-centered under the influence of Confucianism (e.g., Moloney and Xu, 2015; Han and Han, 2019). On the other hand, others reported that the experience of teaching in another culture resulted in CFL teachers’ transformation of professional identity from an authoritative teacher on stage to a guiding facilitator by side (e.g., Han et al., 2019; Han and Ji, 2021).

While there is a growing interest in CFL teachers’ identity, our understanding of this group of teachers’ identity construction from the positioning perspective is limited. Positioning can be simply understood as “assigning an identity position to one’s self or to an other” (Reeves, 2009, p. 36). To explicate identity construction through positioning, reflexive and interactive positioning have often been utilized “in an effort to make sense of how we experience ourselves and how we would like to be understood in order to bring structure to our personal lives” (Soreide, 2006, p. 529). Specifically, reflexive positioning refers to the articulation of a self-identified position by an individual (i.e., self-positioning). In contrast, interactive positioning is assigning identity positions to others (i.e., other-positioning or being positioned).

Han and Ji’s (2021)‘s investigation of CFL teachers’ identity formation and reformation in Australian classrooms is one of the few studies based on the positioning theory. The study found that CFL teachers were deeply influenced by their self-identification and their integration with others in the community. The study also found that insufficient communication between self and others resulted in their positioning crisis. Although there is little research on CFL teachers’ identity from the positioning perspective, the teacher education literature in general has shown that (1) “teacher positioning is relational,” (2) “positioning shapes the agency and professional identities of teachers,” and (3) positioning can be beneficial to learning and teaching (Kayı-Aydar, 2019, p. 82).

Informed by interactive and reflexive positioning, this study attempted to understand how novice CFL teachers were positioned as Chinese language teachers in primary schools in New Zealand and whether such being positioned teacher identities were in line with their self-positioning as Chinese language teachers. If not, how the disparities between being positioned and self-positioning may influence novice CFL teachers’ identity construction as Chinese language teachers in primary schools in New Zealand.

### Teacher identity and affordance

The concept of affordance was first proposed by Gibson (1979, 2014). According to Gibson (2014), “[t]he affordances of the environment are what it offers the animal, what it provides or furnishes, either for good or ill” (p. 119), suggesting that the role of affordances can be either positive or negative. In this regard, affordances can be understood as opportunities and challenges for an action perceived by an agent within an environment. However, perceptions may not always result in actions due to the constraints of contexts or personal willingness. Researchers thus distinguished affordances into four levels: potential, perceived, utilized, and shaped affordances (e.g., Kyttä, 2002; Kordt, 2018). Specifically, “potential affordances exist but are not necessarily perceived by the individual; the individual is aware of perceived affordances but may not use them; utilized affordances result in actions, and shaped affordances emerge because the individual actively influences his or her environment and therefore his or her range of affordances” (Kordt, 2018, p. 136). Apart from the above four levels of affordances, the affordance concept also can be categorized as cultural affordances, social affordances, and cognitive affordances, among others (van Lier, 2004).

Regardless of the different types of affordances, the affordances embedded in the community are the same for all teachers. However, teachers may vary in utilizing affordances “due to disparities in their prior education experiences, knowledge, and understanding, a perceived sense of agency in taking control of teaching, identity recognition, and personal goals in work” (Teng, 2019). For example, Haines (2015) examined two Italian as foreign language teachers’ perceptions of the affordances of computer-mediated communication tools over 14 months. The results showed that the two teachers perceived different learning affordances of the same new tools for the same courses.

Although the concept of affordance has been recognized as an influential ecological approach to teacher education, previous research mainly focuses on technology-assisted language instruction (Thoms and Poole, 2017; Jiang and Zhang, 2019; Huang et al., 2021) and multilingual environments (Aronin and Singleton, 2010; Aronin, 2017). Given that teachers’ identity construction is a process of understanding who they are as teachers through socialization within an environment embedded with possibilities, opportunities, and challenges, it is necessary to extend the affordance perspective to examine teachers’ identity construction. Therefore, this study, apart from the positioning perspective, also examined how affordances perceived by novice CFL teachers could contribute to their identity construction as Chinese language teachers in primary schools in New Zealand.

## Methodology

### Research context and participants

This qualitative case study was carried out to examine novice CFL teachers’ identity construction as Chinese language teachers in primary schools in New Zealand from positioning and affordance perspectives. Seven pre-service CFL teachers from China were randomly recruited for the present study. The seven participants were all novice female CFL teachers. According to the literature, teachers with less than 5 years of teaching experience fall in the novice category (Karlberg and Bezzina, 2022; Sun et al., in press). These novice CFL teachers worked as mandarin language assistants in primary schools in New Zealand. The Mandarin Language Assistants is a joint language program launched by the Office of the Chinese Language Council International (Hanban) in China and the Confucius Institute at the University of Auckland in New Zealand. Although mandarin language assistants teach Chinese as a full-time job in New Zealand, they are not eligible to be employed as full-time teachers without a local teacher certificate. Therefore, when mandarin language assistants teach Chinese, a full-time teacher should stay in class for supervision. Table 1 shows the detailed background information of the participants.

**Table 1:** Background information of novice Chinese as a foreign language (CFL) teachers.

Name	Gender	Education background	Prior CFL teaching experience
Ruby	Female	MA in TCSOL	1.5 years in China
Jane	Female	MA in TCSOL	1 year in Hungary
Olivia	Female	MA in TCSOL	1 year in China
Alice	Female	MA in TCSOL	1.5 years in China
Emma	Female	MA in TCSOL	1.5 years in Mexico
Helen	Female	MA in TCSOL	3 years in China
Tina	Female	MA in TCSOL	1 year in Thailand

TCSOL = Teaching Chinese to Speakers of Other Languages.

To become mandarin language assistants in New Zealand, candidates should hand in their application to Hanban. Hanban then selects strong candidates for final interviews. All the successful candidates are required to attend one to two months of training to equip them with essential knowledge and skills for teaching and living abroad. When candidates arrive and settle down in New Zealand, they will be informed by the Confucius Institute in terms of which school or schools they are going to teach. In New Zealand, primary school teachers teach a wide range of subjects such as arts, English, maths, and science in their own fixed classrooms. Given that mandarin language assistants are not fully registered teachers in New Zealand, they do not have their own classrooms for Chinese language teaching. Instead, they have to move between different classrooms with classroom teachers sitting in class to help classroom management if necessary.

### Data collection

Data were collected through retrospective semi-structured interviews after obtaining ethical approval and participant consent. The seven participants were interviewed individually according to their schedules. The interviews ranged from 60 to 80 minutes. During the interviews, the first author asked casual questions to establish a rapport with the participants first. Afterwards, the first author asked a wide variety of open-ended questions designed to elicit information on how they positioned themselves and how they were positioned by others, as well as what external resources, support, and challenges influenced their identity construction in primary schools in New Zealand (see Appendix 1).

The interviews were organized chronologically. The first section collected the early experiences of the participants in order to determine what led them to the CFL teaching career. The second section focused on their positioning and affordances during their Chinese language teaching in primary schools in New Zealand. The third section centered on how their positioning and affordances influenced their identity construction as Chinese language teachers. The last section of the interview elicited the participants’ future commitments. All the retrospective semi-structured interviews were carried out at the end of their first-year teaching in New Zealand. The interviews were conducted in the participants’ native language (Chinese) in order to elicit in-depth information about their “experiences, memories, and their developing professional selves” (Alsup, 2005).

### Data analysis

After the data collection, the first author transcribed all the audio-recorded interviews and returned the transcripts to the participants for their verification and clarification. The transcripts were then analyzed line by line by the second author through a systematic qualitative inductive approach (Corbin and Strauss, 2007). Initially, a total of 260 unique codes were generated across the seven interview transcripts. These codes were then discussed, refined, and reduced to 50 main codes. Based on the narratives of the main codes, they were then grouped into 13 categories, representing five broad themes, including reasons for CFL teaching, self-positioning, being positioned, affordances, and future selves (see Appendix 2 for the coding system). Lastly, we sorted out how these novice CFL teachers constructed their identity as Chinese language teachers in primary schools in New Zealand evidenced by the coded narratives from positioning and affordance perspectives.

To enhance the trustworthiness of the data analysis, the rest authors double-checked the extracted codes, categories, and themes by the second author. When disagreement arose, the authors engaged in a discussion to reach a consensus. Since the transcripts were in Chinese, the excerpts presented in the findings were then translated into English. To preserve the original idea and avoid potential loss of meaning, the authors carefully examined back and forth the selected excerpts and their English translations.

## Findings

### Social level of being positioned, self-positioning, and affordances

Before joining as mandarin language assistants or Chinese language teachers in New Zealand, the participants were all majoring in Teaching Chinese to Speakers of Other Languages (TCSOL) in China. Their identities were “positioned by the society in China as Chinese language teachers serving as a cultural bridge between the East and the West” (*social level of being positioned*, Ruby). Such an identity was further enhanced by the training sessions offered by Hanban for the novice CFL teachers before they were dispatched to New Zealand to teach Chinese (*social level of affordance*). For example,

I have learned much cultural stuff from Hanban’s training, such as tea culture, martial arts, and paper cutting. The training is quite informative. I do not think I would have had the opportunity to learn these skills and knowledge if Hanban did not provide the training. Because of the training, I consciously try to embed cultural elements in my Chinese language teaching. (*social level of affordance*, Ruby)

Although the cultural bridge or cross-cultural facilitator was an identity attributed to them by the Chinese society, all the novice CFL teachers tended to take up this identity position. As evidenced in the interviews, the participants pointed out that they were “honored to be on the mission not only to teach Chinese language but also to help learners understand Chinese people, Chinese culture, and Chinese society” (*social level of self-positioning*, Tina & Emma).

While Hanban’s training serves as a supportive social affordance for novice CFL teachers in terms of how to teach Chinese abroad, the training has some drawbacks. Firstly, the training is too general to include country-specific sessions. As a result, teachers who majored in TCSOL may find the training superficial. Secondly, the training mainly focuses on how to teach college students Chinese. Consequently, teachers who are assigned to primary or secondary schools may find the teaching theories, approaches, and contents offered by Hanban inappropriate for young learners. As Alice recalled, “the training is very general. In fact, it is not so suitable for New Zealand’s education, but it is better than nothing.” (*social level of affordance*, Alice).

After the novice CFL teachers arrived in New Zealand, the Mandarin Language Assistants program helped the novice CFL teachers find a homestay to support their social and cultural adaptation to the new environment (*social level of affordance*). Although homestay, to some extent, enhanced the cross-cultural communication between the novice CFL teachers and the local family, it made some of them feel self-conscious. For example,

The family hosts not only me but also an American. Because they [the host family and the American] share similar cultures and use the same language, they can talk to each other for a long time. Normally, I just keep quiet and listen to their conversation most of the time. I do not know what to talk about and what to share with them. This is a problem for me and the biggest challenge in my life. I want to improve it but feel hopeless. (*social level of affordance*, Alice)

Apart from homestay support, the Mandarin Language Assistants program also organized two training sessions for novice CFL teachers, namely pre-service training and mid-term training (*social level of affordance*). In the pre-service training, the program introduced the education system and teaching mode of New Zealand to the novice CFL teachers. In the mid-term training, the program gathered the novice CFL teachers together for discussion and reflection on their Chinese language teaching in New Zealand. According to the novice CFL teachers, they found the former training “informative” and the latter training “beneficial but insufficient” (*social level of affordance*, Tina).

I find the two training sessions helpful and necessary. The pre-service training provides us with an overview of New Zealand education and shows us the local teaching approaches and styles. It helps me quickly adapt to the local education system and become a local teacher here. The mid-term training offers us an opportunity to discuss teaching difficulties and share effective teaching techniques with each other. However, the mid-term training is only once a year. More sessions of such kind can help us become more competent Chinese language teachers. (*social level of affordance*, Tina)

### Institutional level of being positioned, self-positioning, and affordances

During their teaching in primary schools in New Zealand, the novice CFL teachers realized that they were positioned by schools as language teaching assistants rather than regular teachers, given that they were not registered teachers in New Zealand (*institutional level of being positioned*). According to the regulations of the New Zealand Ministry of Education, only those who have obtained local teaching certificates are qualified to teach in primary or secondary schools in New Zealand. In other words, these novice CFL teachers cannot teach alone until licensed to teach in New Zealand. Therefore, when they teach in schools, a local teacher should stay in class for supervision. Institutionally being positioned as “teaching assistants” or “illegitimate teachers,” to some extent, shaken the novice CFL teachers’ Chinese language teacher identity construction in primary schools in New Zealand. For example,

I am confused about my position in my school as a teaching assistant or a teacher. The school may be unconcerned with my status [position], but the ambiguity of my position in the school affects my mood. It makes me question whether I am a member of this school and whether I am entitled to the same rights of expression and so forth. (*institutional level of being positioned*, Alice)

Although the novice CFL teachers felt perplexed due to being positioned by the schools as assistants, outsiders, and illegitimate teachers, the teachers challenged this idea and positioned themselves as professionally trained Chinese language teachers. As Helen recalled, “I am no less valued than the local teachers. I am a professional Chinese language teacher. What I teach will benefit children’s future growth” (*institutional level of self-positioning*, Helen). Some novice CFL teachers also “believed that [their] efforts in Chinese language teaching in schools have changed the schools’ positioning of [them] from being assistants to teachers” (*institutional level of self-positioning*, Emma).

In terms of the institutional level of affordances, “schools normally arrange classroom observations for [the novice CFL teachers] to help [them] get the first-hand understanding of the local teaching environment and classroom features” before they take their role as Chinese language teachers (*institutional level of affordance*, Helen). The pre-service observation is usually one-week long. Through this affordance, novice teachers can learn from experienced teachers the methods of classroom management and familiarize themselves with the learning habits, styles, and characteristics of students with different ethnic backgrounds. As Olivia recalled, “observing local teachers’ classes helped me a lot because I gained an understanding of what strategies I can use to educate the local students, to attract their attention, and to maintain discipline in class” (*institutional level of affordance*, Olivia).

Schools also offered morning tea breaks, allowing teachers to mingle and build rapport (*institutional level of affordance*). Nevertheless, most novice CFL teachers felt awkward during the breaks. As some recalled, “when I am in the staff room for a morning tea break, I do not know what I can talk to my colleagues. I do not know how to chat with them” (*institutional level of affordance*, Alice). The local teachers, on the other hand, “just think there is a new colleague. They do not pay attention to you” (*institutional level of affordance*, Tina). “They just do not care to talk to you” (*institutional level of affordance*, Helen).

Although classroom observations and morning tea breaks were offered to help the novice CFL teachers adapt to the new environment, the lack of standardized curriculum and syllabus for Chinese language in New Zealand (*institutional level of affordance*) left these teachers feeling vulnerable. With no reference for Chinese language teaching, these novice CFL teachers had to design their own lesson plans and teaching materials. The struggling situation, to some point, waned these teachers’ self-efficacy and made them anxious about deciding what and how to teach Chinese to their students. For example,

There is no textbook or curriculum I can refer to, and there is no one I can talk to in terms of Chinese language teaching. I am the only Chinese teacher in my school. This is the case for most schools. Without a curriculum or a syllabus as a guide, I am a bit lost. I have to design my lesson arbitrarily depending on my own preferences. It would be better if there were any achieved documents for reference (*institutional level of affordance*, Jane)

### Individual level of being positioned, self-positioning, and affordances

Teacher identity is established through negotiating different positions (Huang and Wang, 2021). In the case of these novice CFL teachers, how they positioned themselves along with how they were positioned by other individuals such as teachers and students would shape who they were as teachers in New Zealand. For example, the local teachers, following the schools’ position on the novice CFL teachers, did not regard them as “colleagues but temporary helpers” (*individual level of being positioned*, Ruby). As Olivia pointed out, “I do not think my colleagues see me as a regular teacher but rather a trainee teacher. They are unwilling to engage in deep conversations. To them, I am a random person who will go back to China after just one or two years” (*individual level of being positioned*, Olivia). Similarly, other novice CFL teachers found that they were positioned by the school as “tools” and “teaching assistants” (*individual level of being positioned*, Emma).

Although being positioned by their colleagues as temporary helpers rather than teachers was discouraging, the novice CFL teachers did not lose their identity as professionally trained Chinese language teachers. In effect, they were proud to be “Chinese teachers” and identified themselves as a “cultural bridge” (*individual level of self-positioning*, Tina) and a “cross-cultural promotion ambassador” (*individual level of self-positioning*, Emma) between China and New Zealand. As Jane and Emma pointed out, “through our teaching, students may understand Chinese language, culture, and people better. It is meaningful to teach Chinese abroad” (*individual level of self-positioning*, Jane). “The profession [as a Chinese language teacher] is a great cause to introduce and promote Chinese language and culture worldwide. I am eager to attract more people to learn Chinese” (*self-positioning*, Emma).

Apart from colleagues, students also played a role in the novice CFL teachers’ self-positioning. Specifically, the novice CFL teachers expressed that they were respected and liked by their students. This, in turn, “motivated [them] to commit more to [their] Chinese language teaching” (Tina). The above positive relationship enabled the novice CFL teachers to “gain a great sense of accomplishment and satisfaction from being a Chinese language teacher” (*individual level of self-positioning*, Ruby). These novice CFL teachers were also positioned as friends by their students over time due to their strengthened bond. As Alice recalled, “I am more than a teacher to my students now. I am a friend in my students’ eyes who can play with them and teach them some Chinese” (*individual level of being positioned*, Alice). However, the friend identity caused classroom management issues for the novice CFL teachers, as “students may not follow instructions accordingly” (*individual level of affordance*, Alice).

## Discussion, implications, and conclusion

This study examined seven novice CFL teachers’ identity construction in an overseas context in an attempt to understand how positioning and affordances could contribute to the novice CFL teachers’ identity construction as Chinese language teachers in primary schools in New Zealand. In general, the findings of the present study suggest that the novice CFL teachers’ identity can be subject to being positioned, self-positioning, and affordances at social, institutional, and individual levels (see Table 2).

**Table 2:** Novice CFL teachers’ positioning and affordances in primary schools in New Zealand.

Level	Being positioned	Self-positioning	Affordances
Social	• Cultural ambassador by Hanban	• Cultural bridge • Professionally trained Chinese language teachers	• Training by Hanban • Training by the program • Homestay by the program
Institutional	• Outsiders or illegitimate teachers by schools	• Professionally trained Chinese language teachers	• Classroom observation • Tea breaks
Individual	• Teaching assistants or illegitimate teachers by colleagues • Teachers and/or friends by students	• Professionally trained Chinese language teachers	• Classroom management issues

### Double-edged role of positioning in novice CFL teachers’ identity construction

In terms of being positioned in teacher identity construction, it refers to a process of individuals’ being externally assigned positions that may influence who they are as teachers within a particular context. In this study, how the novice CFL teachers were positioned played a double-edged role in their identity construction as Chinese language teachers in primary schools in New Zealand. For example, being positioned as “cultural ambassador” or “cultural disseminator” at the social level motivated novice teachers not only to teach Chinese well but also to embed Chinese culture smoothly into teaching. However, being positioned as outsiders and illegitimate teachers at the institutional and the individual levels, to some point, perplexed the novice CFL teachers’ identity construction as Chinese language teachers in primary schools in New Zealand.

In terms of self-positioning in teacher identity construction, it refers to a process of individuals’ assigning reflexive positions to support who they are as teachers within a particular context. In this study, how the teachers positioned themselves profoundly influenced the novice CFL teachers’ identity construction as Chinese language teachers in primary schools in New Zealand. Specifically, the findings of the study showed that although being positioned as outsiders and illegitimate teachers at the institutional and individual levels, the novice teachers’ consistent self-positioning as professionally trained Chinese language teachers helped them stay motivated to teach Chinese well in order to maintain their image as successful Chinese language teachers.

The above positioning findings add evidence to the literature that self-positioning (i.e., reflexive positioning) and being positioned (i.e., interactive positioning) can help make sense of how different positions experienced by teachers may influence their teacher identity construction (Soreide, 2006). Specifically, our study echoes Han and Ji (2021), showing that the inconsistency between being positioned and self-positioning identities may shake novice CFL teachers’ identity construction. For example, our study revealed that there was a conflict between self-positioning as professionally trained Chinese language teachers and being positioned as outsiders and illegitimate teachers. This conflict led to the identity confusion among the novice CFL teachers.

The above positioning findings also support the literature that positioning is relational; can shape teachers’ agency and identity; and can be beneficial to learning and teaching (Kayı-Aydar, 2019). For instance, in our study, the novice CFL teachers were relationally positioned by colleagues and students. Specifically, being positioned by colleagues as teaching assistants and/or illegitimate teachers inspired the novice CFL teachers to devote themselves to Chinese language teaching. With the recognition of their teaching ability from colleagues and students, the novice CFL teachers consolidated their identity as professionally trained Chinese language teachers. However, being positioned by students as friends, to some extent, resulted in the novice CFL teachers’ inability to manage classrooms well. This challenged these teachers’ identity as professionally trained Chinese language teachers. The above facts corroborate the literature that teachers’ identity construction is contingent on how they position themselves and are positioned by others in their situated contexts (Yazan, 2018).

### Positive role of affordances in novice CFL teachers’ identity construction

In terms of affordances in teacher identity construction, the present study showed that affordances, such as the social level of teacher training and the institutional level of classroom observation, helped the novice CFL teachers adapt to Chinese language teaching in primary schools in New Zealand. This, in turn, enhanced the novice CFL teachers’ identity construction as professionally trained Chinese language teachers rather than teaching assistants or illegitimate teachers in New Zealand. Nevertheless, there were also circumstances that challenged the novice CFL teachers’ identity as professionally trained Chinese language teachers. For example, the lack of the institutional level of affordances, such as referable syllabus and appropriate textbooks, made the novice CFL teachers feel anxious and vulnerable. Additionally, the lack of the individual level of affordances, such as students’ classroom cooperation, led the novice CFL teachers to question their classroom management competence and thus shaken their identity construction as professionally trained Chinese language teachers.

The above affordance findings support Gibson’s (2014) point of view that affordances are what an environment offers for an agent, either for good or ill. In this study, if affordances are opportunities to novice CFL teachers, it is likely to enhance their teacher identity construction. Whereas, if affordances are challenges to novice CFL teachers, it may weaken their teacher identity construction. This echoes the existing literature that teachers may face various opportunities and challenges in the process of their Chinese language teacher identity construction in an overseas context (e.g., Moloney and Xu, 2015; Han and Han, 2019; Han and Ji, 2021; Sun, 2021). Moreover, the present study enriches the literature by extending the affordance perspective to the examination of teachers’ identity construction. Specifically, the study adds to our understanding that affordances can be hierarchically interpreted from social, institutional, and individual levels.

### Implications

To support novice CFL teachers’ identity construction as Chinese language teachers in overseas contexts, schools and teacher training programs may take the following suggestions into consideration. Firstly, it is important to equip novice CFL teachers with solid professional knowledge and diverse teaching skills to help them establish concrete self-positioning such as professionally trained teachers. For example, schools and teacher training programs may consider opening a course tailored for prospective CFL teachers in terms of how to teach Chinese overseas. Schools and teacher training programs may also consider inviting veteran CFL teachers to share their overseas teaching experiences and tips to strengthen their overseas teaching ability.

Secondly, it is important to create an inclusive and welcoming environment (Chu et al., 2021; Liu and Chu, 2022) for novice CFL teachers to make them feel being positioned as insiders rather than outsiders. For instance, host schools are recommended to introduce newcomers of CFL teachers to the school staff through emails, meetings, and school websites. Host schools are also recommended to set up a fixed classroom for CFL teachers instead of having them run from one classroom to another to teach Chinese. All of the above may make the newly arrived CFL teachers feel welcomed, valued, and supported as insiders in their host schools.

Lastly, it is advised to enhance novice CFL teachers’ awareness of affordances so as “to enable them to profit maximally from the resources at their command” (Singleton and Aronin, 2007, p. 83). Specifically, teacher training programs may inform novice CFL teachers in terms of what resources and materials they can utilize to tailor their Chinese language teaching for local students. Moreover, host schools may invite novice CFL teachers to reflect on cases when there are insufficient affordances or too many challenges in the process of their Chinese language teaching. As a result, schools may adjust their affordances accordingly to better support novice CFL teachers’ classroom teaching and thus their Chinese language teacher identity construction.

### Conclusion

Summing up, this study reports on the novice CFL teachers’ identity construction in primary schools in New Zealand from an integrated lens of positioning and affordance. The findings show that the novice CFL teachers’ identity construction can be subject to the social, institutional, and individual levels of being positioned, self-positioning, and affordances. Specifically, (1) consistent self-positioning at the social, institutional, and individual levels could, to a large extent, determine the novice CFL teachers’ identity construction; (2) inconsistency of identities between being positioned and self-positioning at the social, institutional, and individual levels might weaken the novice CFL teachers’ identity construction; and (3) affordances as opportunities at the social, institutional, and individual levels could strengthen the novice CFL teachers’ identity construction, whereas affordances as challenges could not.

## Limitation and future research

There are some limitations of the present study. First, the participants of the study are all females with a similar educational background. The findings of the study, therefore, may not be generalizable to other samples. Future research may consider examining other types of CFL teachers, such as novice in-service teachers, experienced in-service teachers, and non-TCSOL major teachers, to obtain a more comprehensive understanding of how positioning and affordances may influence CFL teachers’ identity construction. Second, the findings of this single sourced qualitative study may not be convincing. Other qualitative data collection methods, such as focus groups and naturalistic observation, could have been adopted to cross-validate the findings of the present study. Lastly, this study focuses on novice CFL teachers’ identity construction from positioning and affordance perspectives. Future research is advised to carry out comparative studies to investigate whether there are any disparities between different types of CFL teachers’ identity construction as Chinese language teachers in an overseas context.

## Data availability statement

The datasets presented in this article are not readily available because the raw data supporting the conclusions of this article may be requested from the first author. Requests to access the datasets should be directed to PS, luapnus@zju.edu.cn.

## Ethics statement

The studies involving human participants were reviewed and approved by the University of Auckland Human Ethics Committee (014726). The patients/participants provided their written informed consent to participate in this study.

## Author contributions

PS contributed to the data collection, data analysis, and manuscript writing. YW contributed to data analysis and manuscript writing. YL contributed to the manuscript review. ZL contributed to the data collection. All authors contributed to the article and approved the submitted version.

## Funding

The study was supported by the Research Project on International Chinese Education (Grant no. 20YH05D) and the Fundamental Research Funds for the Central Universities in China.

## Conflict of interest

The authors declare that the research was conducted in the absence of any commercial or financial relationships that could be construed as a potential conflict of interest.

## Publisher’s note

All claims expressed in this article are solely those of the authors and do not necessarily represent those of their affiliated organizations, or those of the publisher, the editors and the reviewers. Any product that may be evaluated in this article, or claim that may be made by its manufacturer, is not guaranteed or endorsed by the publisher.
