# Supplementary material for: Novice Chinese as a foreign language teachers’ identity construction in primary schools in New Zealand from positioning and affordance perspectives
Source: Front Psychol. 2022 Nov 10;13:979803. doi: 10.3389/fpsyg.2022.979803 (PMC9706993; doi:10.3389/fpsyg.2022.979803)
Supplement: Supplementary file 1 [file Data_Sheet_1.doc]

Appendix 1. Retrospective Semi-structured Interview Protocol

*Section 1 background information*

- Can you briefly introduce yourself, including your name and your educational background?
- How did you end up being a Chinese language teacher in New Zealand?
- Did you have any Chinese language teaching experience before coming to New Zealand? If yes, can you share the experience with us?
- Did you experience any difficulties while teaching Chinese? If yes, how did you overcome the difficulties?

*Section 2 positioning and affordances*

- Is being a Chinese language teacher a well-recognized job by society in China?
- Before teaching Chinese here in New Zealand, did Hanban or your home school provide any training for you in terms of how to teach Chinese abroad?
- After you arrived here, did the Confucious Institute or the host school provide any support for your Chinese language teaching in primary schools here? If yes, were you happy with the support? Why and why not?
- Do you think you are a regular teacher or just an assistant in your host school? Why? Any stories?
- What is your own recognition of yourself? A Chinese language teacher or a mandarin language assistant? Why?
- Do the local teachers regard you as colleagues? Why or why not?
- Do you agree with these local teachers’ recognition of you?
- What about your students? What is your image in their mind?
- Are you happy with your students’ perception of you?
- Have you experienced any challenges or difficulties during your Chinese language teaching in primary schools in New Zealand? If yes, how did you cope with it?
- Have you tried to blend in the local school? Any stories?

*Section 3 influences of positioning and affordances*

- Do you think that the public’s opinion will influence your decision to become a Chinese language teacher in New Zealand? Why and why not?
- What encouraged you to become a Chinese language teacher here?
- What is your host family experience like? Is it helpful for you?
- Did Hanban, the host school, or your colleagues help you when you encountered difficulties in Chinese language teaching? And how?
- How do you normally socialize with your colleagues? Are you happy with it?
- Are you satisfied with what you have been perceived by your host school, your colleagues, or your students? Why?
- Have you experienced any identity crises in school? How did you solve it?

*Section 4 future commitment*

- What is your plan for your future career?
- Do you foresee working as a Chinese language teacher here or somewhere else?

Appendix 2 A Sample of Coding System Informed by Positioning and Affordance

| Theme | Category | Code | Example |
| --- | --- | --- | --- |
| CFL teaching reasons | Internal reasons | Interest | I like teaching Chinese to foreigners. It is interesting to meet people from different counties. (Ruby) |
|  |  | Achievement | I think it has a lot to do with the sense of achievement. I will feel proud of myself when my students become fluent in Chinese. (Emma) |
|  |  | Cultural exchange | I have always wanted to become a cultural bridge between different countries. Teaching Chinese provides me with such an opportunity. (Olivia) |
|  | External reasons | Exotic experience | Through Chinese language teaching programs, I am able to visit different countries and experience different cultures. (Helen) |
|  |  | Long holidays | One of the advantages is that you can enjoy long holidays compared with other jobs. (Ruby) |
| Self-positioning | Social level | Cultural bridge | I am honoured to take on this position as a cultural bridge between China and New Zealand to help people understand each other better. (Tina) |
|  |  | Cross-cultural facilitator | A CFL teacher means cross-cultural communication to the public. (Jane) |
|  | Institutional level | Professional CFL teachers | Although I am not fully regarded as an insider but just a mandarin language assistant in the school, I am no less valued than the local teachers. I am a professional Chinese language teacher. What I teach will benefit children’s future growth. (Helen) |
|  |  | Position changed | My efforts in Chinese language teaching in schools have changed the schools’ positioning of me from being assistants to teachers. (Emma) |
|  | Individual level | Cultural bridge | I define myself as a cultural bridge. Through my teaching, students may know more about Chinese language, culture, and people. (Jane) |
|  |  | CFL teacher | I gain a great sense of accomplishment and satisfaction from being a Chinese language teacher. (Emma) |
| Being positioned | Social level | Cultural bridge | I think we are positioned by the society in China as Chinese language teachers serving as a cultural bridge between the East and the West. (Ruby) |
|  |  | Cross-cultural facilitator | We are expected by Hanban to become cultural facilitators between different countries. |
|  | Institutional level | Illegitimate teachers | We do not have the local teacher certificate. So, we are illegitimate teachers in our schools. Technically, we are not allowed to teach here. (Olivia) |
|  |  | Outsiders | I think my school regards me as an outsider, someone who will leave in a year or two. (Alice) |
|  | Individual level | Assistants by colleagues | My colleagues did not regard me as a colleague but more like a temporary helper to them. (Ruby) |
|  |  | Friends by students | I am more than a teacher to my students now. I am like a friend in my students’ eyes who plays with them and teach them some Chinese. (Alice) |
| Affordances | Social level | Opportunity | I have learned much cultural stuff from Hanban’s training, such as tea culture, martial arts, and paper cutting. The training is quite informative. (Ruby) |
|  |  | Challenge | However, the mid-term training is only once a year. More sessions of such kind can help us become more competent Chinese language teachers. (Tina) |
|  | Institutional level | Opportunity | Observing local teachers’ classes helped me a lot because I gained an understanding of what strategies I can use to educate the local students, to attract their attention, and to maintain discipline in class. (Olivia) |
|  |  | Challenge | When I am in the staff room for a morning tea break, I do not know what I can talk to my colleagues. I do not know how to chat with them. (Alice) |
|  | Individual level | Opportunity | There was a Maori teacher who was very kind. He offered some suggestions about how to be a good teacher. (Emma) |
|  |  | Challenge | Students may not follow my instructions well because they do not treat me like a teacher. (Alice) |
| Future selves | Teachers | CFL teacher | I like teaching Chinese I think I will continue to be a Chinese language teacher. (Jane) |
|  | Non-teachers | Book editor | If I cannot continue to be a Chinese language teacher, I may become a child book editor. (Helen) |
